# Human resilience depends on distinctively human brain circuitry and development

**DOI:** 10.3389/fnbeh.2024.1370551

**Published:** 2024-05-16

**Authors:** Mark Reimers

**Affiliations:** Institute for Quantitative Health Sciences and Engineering, Michigan State University, East Lansing, MI, United States

**Keywords:** resilience, prefrontal cortex, brain development, plasticity, depression

## Abstract

Most studies of psychological resilience in the past century have focused on either biological or social psychological correlates of resilience or depression. This article argues that the two approaches need to be integrated because of uniquely human processes of cortical development during early childhood. The article concludes with some suggestions for integrative research agendas.

## 1 Introduction

Understanding psychological resilience is becoming an urgent issue, as diagnoses of anxiety and depression have skyrocketed over the past generation in the Western world and Global South, despite increased attention to mental health in schools and the workplace. So far, researchers have mostly studied the phenomenon of psychological resilience, either in relation to life experience (especially social relationships), or in terms of physiology or molecular biology. Researchers agree that both factors matter but these different aspects of resilience seem to inhabit different conceptual worlds and are rarely discussed or studied together.

The aims of this perspective are:

(i) To briefly review research into contributions to resilience from life history (“nurture”) and genetics (“nature”) and to argue that genetic evidence points to the importance of life history.
(ii) To draw attention to some distinctive, but poorly known, aspects of the human brain, by which early experience could modulate physiology, in ways different from rodent, and even primate, models of resilience.
(iii) To suggest opportunities for resilience research that integrate biology with social experience.

These observations may also shed some light on the heterogeneous ways in which humans may be resilient; and on why many effective interventions with rodent models of depression have not translated into consistently effectively human therapies.

Many authors have noted that the concept of resilience is poorly defined and the usage of the word has changed in the short history of systematic psychological research. Researchers still argue over whether to think of resilience as trait or phenomenon. Vernon (2004) summarizes the intellectual history of the idea of resilience in psychology. In the early and mid-20th century, “resilience” seems to mostly refer to people recovering from the horrors of war. According to Google Ngram, the use of the word “resilient,” in contexts related to psychological trauma (e.g., “mental resilience” and “resilience of mind”), rose during the First World War and peaked in the early 1960s. During the 1950s and 1960s the word came to be associated more with good mental health and life outcomes in children from high-risk circumstances, such as poverty or abuse. Ngram shows a strong uptick in use of the word “resilient” in a wider sense (e.g., “resilient to trauma” and “emotional resilience”) starting in 1980. By the 1990s the word was being used more often to describe bouncing back from common life stresses, like job loss, a divorce, or a death in the family. This article will use the currently popular sense of resilience as resistance to depression or anxiety after serious adversity, in accord with the American Psychological Association (2023) definition. However, it is worth keeping in mind that the meaning of resilience has changed and is still changing.

## 2 Resilience and early life experience

In the mid-20th century, thought leaders in mental health (such as Sigmund Freud, John Bowlby, and many others) advocated psychodynamic and social-environmental theories to explain psychological distress. Their theories, drawn initially from patient narratives elicited in therapy, emphasized the role of early life experiences, such as childhood trauma, aberrant attachment styles, and dysfunctional family dynamics, in promoting or compromising mental health.

However, the narratives told in therapy could rarely be independently verified and were often resisted by other family members. In 1982, Australian psychiatrist Gordon Parker published a review of the most systematic studies into the early childhood dynamics of schizophrenic patients, finding no consistent association between liability to schizophrenia and early childhood experience (Parker, 1982).

There have been many interpretations of this failure: maybe the observers were not looking at the most relevant aspects of parenting; maybe the theories were wrong; most studies used retrospective reports, liable to memory distortions, and even those studies that used expert judgments – necessarily based on a short interview in controlled conditions – may not reflect the important drivers of family dynamics. However, during the 1980s the Zeitgeist in politics and society shifted away from the environmental determinism of mid-century, and psychodynamic ideas fell out of fashion. A more substantive reason for abandoning these theories was that, as operationalized in mid-century, they did not make good predictions, thus failing a fundamental test of a scientific theory.

## 3 Genes, mental illness, and resilience

The impressive successes of molecular biology and genetics in the second half of the 20th century encouraged scientists to look for molecular explanations of psychiatric phenomena. Early studies showing strong heritability of serious mental illness suggested that genetic studies might easily expose the biology of mental illness. If this were so, the biology of pathology would indirectly illuminate the biology of resilience. However, in 2024, more than two decades on from the Human Genome project, and after more than a thousand gene variants have been implicated in various mental illnesses, genetic evidence has not unraveled the biology of mental illness.

Perhaps the most important finding of behavioral and psychiatric genetics over the past two decades has been that the additional risk or liability for any behavioral disorder or trait conferred by any common variant or single nucleotide polymorphism (SNP) is exceedingly small (Smeland et al., 2020). Although single SNPs have individually small effects, the commonly expressed hope among geneticists in the past decade has that the aggregate effects of many SNPs will enable some accurate predictions. Geneticists compute so-called “polygene scores” by adding many small effects from hundreds of SNPs. However, these polygene scores are not very effective predictors or screening tools for individuals: the most successful example of psychiatric polygene scores to date (Trubetskoy et al., 2022) estimates variance explained by polygene scores at *R*^2^ = 0.07. What this means in practice is that, if one sets a threshold for the schizophrenia polygene score to give a 5% false positive rate, the polygene scores would only identify 13% of the people with a diagnosis (Hingorani et al., 2023). The situation for depression is even murkier: McGeary et al. (2023) recently applied polygene scores from the gold-standard UK Biobank study to out-of-sample population and found predictive *R*^2^ values of 0.03–0.05. No doubt, further research will identify yet more genes, but these genes will have even smaller effects than the ones that we already know and thus it seems unlikely that, in the foreseeable future, researchers will predict depression or resilience from genes alone. Thus, genetic models, on their own, do not seem to make very good predictions, and hence fail the same fundamental test of a scientific theory that (rightly) discredited earlier psychodynamic theories. Something more is needed.

A second important, yet less known, finding is that the majority of common SNPs that are known to contribute to behavioral traits or to psychiatric disorders lie in non-coding, putatively regulatory sections of DNA. This fact was first noted in the landmark study of schizophrenia by the Schizophrenia Working Group of the Psychiatric Genomics Consortium (2014) and found repeatedly since. Recent work has shown that the genetics of behavioral and psychiatric traits are in this way clearly distinguishable from genetic liabilities for neurological disorders, for which more of the heritability lies in coding regions (Reimers and Kendler, 2022).

Consider what this finding implies. Most DNA regulatory regions contain binding sites of proteins that are activated transiently by receptors upon binding their cognate ligand, often a neuropeptide or a small signaling molecule, such as serotonin or dopamine. Neurons and glia express hundreds of receptors for peptides and for small molecule neuromodulators (e.g., serotonin, dopamine, etc.), and most of these signaling cascades induce or repress expression of one or more genes (Jorstad et al., 2023). A SNP in a regulatory binding site may change the affinity of binding, thus changing the production of one or more genes in response to those signals. Thus many signaling pathways within neurons are triggered by electrical activity and neuromodulators, both of which are induced by strong emotion, such as during significant experiences. Thus, these findings suggest that most of the genetic variants that affect risk of psychiatric disorders are those that set the degree of molecular response to emotionally significant events. These findings are consistent with the “orchid/dandelion” hypothesis, that some gene variants increase sensitivity to developmental circumstance (Conley et al., 2013). Thus, even genetics points to the importance of early relationship experience in understanding mental illness or resilience.

## 4 Resilience research needs to incorporate both biology and social experience

Many resilience researchers have recognized the need for “understanding these processes at multiple levels, from genes to relationships” (Masten, 2001). Nevertheless, it has been hard to devise a research program to integrate biology with early relationships. We may hypothesize that early social learning sets pattern for how to modulate attention in social situations and how to assess potential threats or expectation of help.

The next section will outline some specific aspects of human biology that are not often discussed in resilience research, but which underline the importance of integrative research programs. The final section will make some suggestions for potential research programs.

## 5 Human cortical evolution and resilience

Distinctive aspects of human brain physiology and structure are relevant to studies of psychological resilience and depend on both genes and social environment in ways that differ from widely used animal models of resilience or depression. Rodent models are relevant to research on human emotions because the brain stem and basal ganglia circuitry are moderately conserved. Nevertheless, brain circuitry in humans and other primates, especially prefrontal cortex (PFC) circuitry, is well-known to be very differently organized than in rodent models (Laubach et al., 2018). Several authors have drawn attention to some distinctive aspects of the human brain in discussing the gap between rodent models of depression or resilience and human resilience. For example, Alexander et al. (2020) points out that “the wide and persistent translational gap … is due, in part, to a lack of understanding of the complex control of negative affect exerted by the highly evolved primate vmPFC.” However, a comprehensive review of such differences in the context of resilience is yet to be published. Compared to rodents, primates and especially humans, have much more complex inputs to the basal circuitry, which influence recovery from stress or trauma, and which are shaped strongly by early life experience, as will be unpacked below

### 5.1 Prefrontal circuits differ between humans and animal models

Most research on depression and resilience in rodents focuses on ventral tegmental area (VTA), nucleus accumbens (NA), and amygdala. While much of the basic limbic circuitry that links VTA, NA, and amygdala is conserved between rodents and humans, the number and complexity of inputs of input regions has expanded dramatically in primate brains and especially in humans. This prefrontal organization is relevant to studies of depression and resilience because these brain regions and circuits are consistently implicated in depression. For example, sub-genual or ventro-medial PFC (terminology is inconsistent among authors) is consistently found to be over-active in depressed patients (Drevets et al., 2008). Several PFC areas have direct inputs to basal limbic circuitry in primates, and these inputs differ from analogous regions in rodents (Giacometti et al., 2023). Furthermore, the distinctive functions of these input regions seem to differ from their closest rodent analogs. For example, vmPFC has been repeatedly linked to moral expectations and to maintenance of close relationships, such as formed with parents, unlike its rodent analog, infralimbic cortex.

Even compared with monkeys, humans seem to have greatly enhanced inputs from several medial PFC regions to other cortical regions and to amygdala (Giacometti et al., 2023). Recent evolutionary expansions of the uncinate fasciculus and arcuate fasciculus link medial PFC and other parietal and temporal areas in humans more strongly than other primates.

The human brain is so large that conduction delays sending signals across 10 or 15 cm could significantly impair communication between different regions. Primates and other large-brained animals, such as elephants and dolphins, devote more of their brain volume to myelinated axon tracts, but also have many von Economo neurons, which are large neurons with thick axons that transmit signals very rapidly. In great apes and humans, these neurons are especially abundant in the orbital frontal cortex and anterior insula (Cauda et al., 2014). Thus, von Economo neurons quickly broadcast signals from brain areas strongly affected by early social experience (Monninger et al., 2020) to the various association areas, before signals from other similarly remote areas arrive. Comparable pathways that broadcast social brain activity have not been found in rodents.

Although most researchers know that the human brain is three times larger than a chimpanzee’s brain, it is less commonly known that some areas (e.g., sensorimotor cortex) are the same size, and thus relatively smaller in proportion to the rest of the brain, while many areas of the human brain have expanded disproportionately and are relatively larger than in a chimpanzee brain). Among the areas that have expanded greatly in the past few million years are the multimodal association areas (Buckner and Krienen, 2013) and many of the default mode network areas including ventral and medial PFC.

Another recent evolutionary change in the human brain has been the proliferation of dopaminergic interneurons (DIN) in cortex and HC; these are present in humans in numbers typically three times higher than in chimpanzees (Sousa et al., 2017). Little investigation has been done of the specific roles of these DINs in human cognition. However, the physiological effects of dopamine are usually thought to sustain motivation and enhance focused neural activity (Kroener et al., 2009). These DINs are well placed to sustain activity specifically within particular connections and circuits, rather than to affect overall brain activity, as does dopamine from VTA. Thus, we may speculate that the DINs function to sustain activity in specific circuits implementing patterns of emotion and cognition acquired during early life.

### 5.2 Human brain structure is shaped by early experience

The volume of the human brain grows by a factor of four between birth and adulthood. Most growth in human gray matter volume is due to synapses formed in early childhood (Gilmore et al., 2018). In particular, the association areas mentioned above, grow disproportionately in early childhood (Buckner and Krienen, 2013). Studies have shown large individual differences in how these areas are used during common tasks in everyday life (Buckner and Krienen, 2013). Indeed, the development of the visual word form area (“letterbox”) in children who learn to read (Dehaene, 2013), strongly suggest that networks in these association areas are strongly shaped by early childhood experience. Human synapses remain plastic much longer than those of other primates (Liu et al., 2012). Thus the specific patterns of synapse growth fill out the expanding association areas in ways that reflect early life experience, which could explain the large individual differences in how these areas are used in everyday life.

## 6 How we might explore connections between biology and resilience

How can researchers study human resilience, taking into account the biosocial developmental process and the allowing for the large individual variability in ways humans use their brains? We may start by looking to some of the more sophisticated attempts to understand the biology of depression, the opposite of resilience, for some ideas. Several researchers (Buch and Liston, 2021; Williams, 2021), have derived preliminary classifications or dimensions of depression in response to stress, by analyzing patterns of symptoms, or through patterns of activation of various regions observed in fMRI studies. So far, the dimensions identified by these various have not been entirely consistent, although in fairness, the conditions under which brain activity is measured also differ between studies.

Based on the ideas about circuits presented above, perhaps a natural next phase would be to compare early social development, brain structure and patterns of brain activity between resilient and vulnerable people. This could be approached in two complementary ways: retrospectively and prospectively. Retrospective studies could study social brain activity in relation to prior resilience; prospective studies could track development of social brain activity during childhood and measure resilience later on.

First, researchers could identify some people who were resilient to recent adversity and others who were susceptible but are not currently depressed. Both groups could be exposed to naturalistic stresses, such as performing an unsolvable task or resolving a miscommunication. Researchers could track subjects’ attention, using video cameras and modern eye-gaze and facial key-point tracking. Researchers could also measure activation of prefrontal areas and characterize functional connectivity during these tasks, using EEG or fMRI. They could also compare attention and neural activity in response to standardized ambiguous social bids by experimental confederates. These studies could help to identify neural circuits that may mediate resilience in different subsets of people.

A second approach would look at how early social experience affects developing habits of social attention and emotion, together with brain structure and function. Early development is arguably the most likely setting in which to see clearly the distinctive contribution of genes to behavior. Researchers could observe rhythm and texture of naturalistic interactions, such as social bids and responses, motion, and holding (controlling for infants’ alertness and comfort). Researchers could conduct more controlled experimental tests of joint attention by monitoring eye gaze and orienting responses in response to invitations to attention by the experimenter and joint motion or contingent motion of child. Researchers could observe attention and affect in response to social bids by adults or during instrumental use of adults (trying to get adults to do something – e.g., bring over a toy). EEG or fNIRS equipment adapted to infants and children could be used to measure cortical activity, and again, analysts could characterize patterns of usage of cortical association areas and functional connectivity during these tasks.

Such studies are unlikely to have sufficient numbers of subjects for a well-powered genome-wide association study. However, researchers could analyze observed measures of attention and brain activity in relation to the smaller number of genetic loci thought to be involved in mental illness.

## 7 Conclusion

Scientists seek simple and powerful explanations. However, a complex phenomenon like resilience may not yield to a simple explanation. There may be several distinct processes underlying resilience and resilient people may engage more than one such process in any particular situation. These processes seem likely to be shaped by early relationship experience, in ways still to be uncovered. Untangling these processes and the circuits that support them could be a major project for the 21st century.

## Author contributions

MR: Conceptualization, Writing – original draft, Writing – review & editing.
